# The impact of a sterile processing program in Northwest Tanzania: a mixed-methods study

**DOI:** 10.1186/s13756-019-0633-0

**Published:** 2019-11-20

**Authors:** Olive Fast, Faith-Michael Uzoka, Alexander Cuncannon, Christina Fast, Aliyah Dosani, Elias Charles Nyanza, Dan Fast, Theresia Maduka

**Affiliations:** 1 Mount Royal University, 4825 Mount Royal Gate SW, Calgary, Alberta T3E 6K6 Canada; 2 Mount Royal University, University of Calgary, O’Brien Institute for Public Health, Calgary, Canada; 3 Sterile Processing Education Charitable Trust, Calgary, Canada; 4 Mount Royal University, University of Calgary, O’Brien Institute for Public Health, Calgary, Canada; 5 Catholic University of Health and Allied Sciences, Bugando, Mwanza, Tanzania

**Keywords:** Sterile Processing, Decontamination, Sterilization, CSSD, Safe Surgery, Tanzania, Education, Mixed-methods, Mentoring

## Abstract

**Background:**

Inadequate training of health care workers responsible for the sterilization of surgical instruments in low- and middle-income countries compromises the safety of workers and patients alike.

**Methods:**

A mixed methods research study was initiated in the Lake Zone areas of Northwestern Tanzania in the summer of 2018. The goal was to identify the impact of education and training on sterile processing practices at ten hospitals. Quantitative data analyzed included hospital assessments of sterile processing practices prior to and 4 months after training, as well as participant test scores collected at the beginning of training, after 5 days of classes, and 4 months after mentorship was completed. Thematic analysis of interviews with participants 4 months post-training was completed to identify associated impact of training.

**Results:**

Improvement in test scores were found to be directly related to sterile processing training. The greatest sterile processing practice changes identified through hospital assessments involved how instruments were cleaned, both at point of use and during the cleaning process, resulting in rusted and discoloured instruments appearing as new again. Themes identified in participant interviews included: changes in practice, challenges in implementing practice changes, resource constraints, personal and professional growth, and increased motivation, confidence and responsibility.

**Conclusions:**

Providing education and follow up support for workers in sterile processing resulted in increased knowledge of best practices, application of knowledge in practice settings, and awareness of issues that need to be overcome to decrease risks for patients and health care workers alike. Further research is needed to identify the impact of mentorship on hospital sterile processing practices in order to provide clear direction for future spending on training courses.

## Background

The Lancet Commission found that 81 million individuals “become impoverished seeking and receiving surgical care” [1]. Lack of access to surgery for many leads to loss of income as they are unable to work or care for their families due to extended illness, permanent debilitating injury or even death. To strengthen global health and increase health coverage, improved surgical services are needed. However, current resources are limited. Studies conducted in low- and middle-income countries (LMICs) indicate that the incidence of surgical site infections (SSIs) are higher than in high income countries, citing rates from 10.9 to 70% [2–4]. These studies link SSIs to equipment and instruments that are “often unusable or only partly usable owing to a lack of resources for maintenance or replacement” (p. 17) [5]. Shah et al. (2012) [6] note that SSIs are the most common healthcare associated infection, the main cause of which is the entrance of a microorganism during surgical intervention. Key areas have been identified in research papers for addressing infection prevention and control issues in African hospitals. These include: introducing appropriate infection control teaching for health care workers (HCWs), improving basic hygiene, isolation precautions, sterilization and waste disposal; promoting good infection control practices related to hand cleaning, dressing techniques and surgical procedures; identifying HCWs with specific responsibility for infection control; and developing surveillance networks to increase data on facility infections [7].

## Methods

This mixed-methods research studied the impact of a sterile processing (SP) training course program provided for HCWs from 10 Tanzanian hospitals in 2018 by a charitable not-for-profit organization, Sterile Processing Education Charitable Trust (SPECT). Quantitative data collected involved hospital assessments of SP practices using a Hospital (SP) Assessment Form (See Additional file 1) both prior to training and again at 4 months post mentoring visits. Safe Surgery 2020 (SS2020) [8] hospital leaders were invited to send 2 to 5 HCWs directly involved in reprocessing practices or responsible for overseeing SP practices for a 5 day training program. All participants completed a test (See Additional file 2) at the beginning and end of the training and again 4 months post mentoring. SP pre- and post-knowledge tests were administered, and facility assessment data were collected to determine retention of learning and implementation of changes in practice.

Of those who attended the training, 2 to 5 HCWs from each facility were selected for a 2 day Training of Trainers (ToT) workshop train the trainer sessions. Selection was based on engagement in the theoretical component provided during the 5 days prior. Following the classroom component of the program, SPECT's educator visited each hospital twice, mentoring participants at their work sites to support them and provide consultation services regarding on-site SP practices.

Qualitative data included 20–30 min semi-structured interviews (See Additional file 3) conducted 4 months post mentoring to determine participants’ perceptions of the impact education and mentorship had on their practice. Interviews were conducted by the research assistant (TM) and recorded, then transcribed and translated from Swahili to English. Participants are quoted using their identification number and regions are identified alphabetically. Data collection began March 2018 and concluded in January 2019.

### Data analysis

Pre- and post-education and mentorship data, as well as facility SP assessments, were compared to identify changes in practice and knowledge acquisition. Quantitative data from participant knowledge tests were analyzed with the IBM Statistical Package for Social Sciences (SPSS) Statistics 23 software, using the Wilcoxon paired test. To examine the hypotheses that the treatments (training) had significant effects on the participants, we conducted non-parametric tests using Wilcoxon and Friedman statistics. In both tests, the improvements were considered statistically significant if the *p*-value ≤0.05. Clinical significance of our results was also determined using Cohen’s d described in Fast et al., 2019.^9^

## Results

In both regions the results of the Wilcoxon test indicate that increases in SP knowledge of HCWs for the first and second tests respectively were because of the training received (Additional file 3: Table S1). We also observed that the effect in region A hospitals was significantly higher than the effect in the region B hospitals. Clinical effectiveness of treatment based on the first and second post-tests indicate there was an aggregate drop in effectiveness of 10.53% between the first and second post-tests for region A hospitals. Region B hospitals recorded an aggregate drop of 40.53%. To determine the overall (steady state) effect of the training we conducted a Friedman test on the combined post-training test data (post-train 1 and 2). The results were also found to be statistically significant for each of the two regions.

### Analysis of hospital assessment findings (Additional file 4)

#### Point of use preparation

The greatest improvements in point of use preparation were the wiping of visible blood from contaminated instruments (Pre 1; Post 9) and soaking of instruments in water and detergent after use (Pre 1; Post 8).

#### Transport of items to decontamination area

Flow of instruments from dirty to clean areas during transportation proved difficult for many of the areas (Pre 3; Post 5), as the structure of the facility and locations of the areas often did not accommodate a one-way flow to decrease the risk of cross-contamination. To mitigate this problem, participants were instructed to cover contaminated instruments during transport, and on post assessment it was noted that nine areas were following this practice, where only one area had been covering instruments during the pre-training assessment.

#### Manual cleaning of instruments

The most substantial change in the cleaning process was the elimination of 0.5% sodium hypochlorite solution to decontaminate instruments after use in 8 of the 13 areas, and those that still used it paid attention to limiting the immersion of instruments to less than 10 minutes. As SPECT had provided all participants with toothbrush-sized brushes for manually cleaning surgical instruments, 11 areas used these brushes for cleaning purposes, whereas only one area had access to appropriate brushes prior to training.

#### Inspection, assembly & packaging in clean area

Inspection of instruments prior to packaging, to ensure they were clean and functional, went from 10 areas to 13 areas post-training, while two more areas moved instruments away from the dirty area for package assembly (Pre 10; Post 12). Participants were taught to let instruments air dry if they did not have clean, lint free clothes available. Thus, fewer areas hand dried instruments post-training (Pre 7; Post 5). There was also an increase in the practice of ensuring hinged and ratchet instruments were placed in open and unlocked position during assembly (Pre 7; Post 11) to ensure full contact with steam during the sterilization cycle.

#### Sterilization

The largest improvement in the sterilization process was the use of chemical indicator tape placed on the outside of packages (Pre 5; Post 10) for visual assurance that items opened in the operating theater had gone through the sterilization process. To further verify that steam had reached the instruments, 6 hospitals were using folded pieces of indicator tape or type five internal chemical integrators donated by SPECT inside instrument packages post-training, whereas none had done so pre-training.

#### Sterile storage

In 3 areas, sterilized instrument sets were moved from open spaces to enclosed cabinets for storage post-training, to protect packages from contaminants prior to use (Pre 8; Post 11).

### Thematic analysis of participant interviews (Additional file 5)

Several themes were identified through qualitative analysis that highlighted successes of the training, challenges in implementing practice changes, and issues that arose as a result of the training. Participants most frequently commented on the following five themes, from greatest to fewest: changes in practice, challenges in implementing practice changes, resource constraints, personal and professional growth, and increased motivation, confidence and responsibility. Other themes were noted to occur less frequently, but were still relevant to our findings, including: influence of formalized policy and guidelines, reciprocal relationship and partnership building and perceived changes in SSI incidence rates.

## Discussion

Findings in this study support evidence of SP practice change from a short training program presented in similar studies in Benin and Ethiopia [9, 10]. While participants showed retention of knowledge 4 months post-training that was attributable to SPECT’s training, knowledge retention was noted to be higher in region A than region B. An evaluation of interviews was undertaken to account for the difference in retention. It was observed that in participant interviews, substantially more participants in region A spoke about infection control guidelines and policy. Greater resistance by administration in region A to practice changes was noted. It is surmised that these factors may have increased participants need to articulate evidence for practice change, thereby reinforcing their own learning and increasing knowledge retention of SP. Knowledge increase post training in both the Benin and Ethiopia study indicated knowledge retention, but did not evaluate its clinical significance. Our analysis shows that SPECT’s training was clinically significant (Additional file 3: Table S2).

Hospital assessments of each area indicated improvements in many aspects of the SP practice, most significantly the cleanliness and functionality of instruments. Similar to the Benin and Ethiopia studies these changes resulted in ability to perform safer surgery due to increased instrument functionality and sterility of instruments. Of particular note is that participants learned without proper cleaning it is impossible to sterilize instruments – a truth they had not previously understood.

The most significant impact of the training identified by participants was that the instruments were noted to be clean and functional. This positive effect was a result of several improvements in SP practice, including the removal of 0.5% sodium hypochlorite solution to decontaminant used surgical instruments, access to and use of brushes and rust remover, as well as SP workers’ attention to inspection and function testing of the instruments prior to packaging. “Chlorine was really destroying instruments. Even now many instruments are functioning well, they don’t have rust.”A12.

Because of the clean and sterile instruments, and increased attention to sterile processing practices, surgical site infections were noted to be decreased.

“Before the training sepsis rates were very high. After a procedure a woman would come back with open wounds. … Some stakeholders were asking why they do not see a sepsis report and we said we don’t have [any] because patients don’t get gaps in wounds, infections and other things, because of the training we received.” B17

Participants identified an increased motivation to change practice after training. B2 noted: “It is true, we are better now. First you know how to protect yourself, you are safe when you go to sterilize instruments - you go knowing what to do, not as before.”

“I feel good, if you have a problem and are given means to solve it, you must feel good.” A7. Not only did satisfaction of sterile processors increase post training, but with their increased knowledge they were able to improve the surgeon’s ability to perform safe surgery. B14 stated:” I am currently proud of my work frankly because everything is good. I have taught my fellow HCWs and they have accepted the changes.”

Similar findings were noted in Benin and Ethiopia, findings that increase the evidence of the need for further attention to SP practices when working to decrease risk of SSIs.

Numerous challenges were also identified by participants, including resistance to practice changes from colleagues and administrators who had not received training, lack of resources, including personnel and supplies, and structural limitations to improved flow from dirty to clean areas. A significant concern noted by participants involved a disconnect between Tanzanian SP guidelines that had been in place and SPECT training received. The issue was that while Tanzania had developed new infection control guidelines, they had not been distributed prior to SPECT’s training. While SPECT made the new guidelines available to participants and administrators alike, other government departments had not yet received the guidelines, specifically the Hospital Accreditation Department. A2 noted: “[We are restricted] from implementing no use of chlorine because once it is inspection season … all places [will] prepare chlorine … because they are things that marks are provided for, so if we tell them about soap they will deduct marks.” While the loss of marks during inspections prevented some administrators from supporting changes to practice, other administrators saw the benefit of the training and noted that a decrease in SSI incidence post-training was evidence enough to support the practice.

## Conclusion

The impact of a SP program in Tanzania has been identified in this paper. As in other areas of healthcare, practice is constantly changing in efforts to improve patient outcomes. Ensuring HCWs are supported to improve their SP practices with education and training is a key step in supporting safer surgery. Further research, however, needs to be done on the difference on-site mentorship has on HCWs as opposed to simply providing education in the classroom. If the focus is on providing education only, not supporting HCWs to identify solutions to complex issues in their setting, then fewer positive impacts may be identified in future. Increasing the focus on surgical support systems, such as SP and related infection control practices, needs to be part of any safe surgery initiative in LMICs to keep patients and HCWs safe.

## Supplementary information

**Additional file 1.** SPECT Sterile Processing (SP) Facility Assessment Tool.

**Additional file 2.** SPECT – Participant Test.

**Additional file 3. **Statistical Analysis. **Table 1.** Hypotheses Testing. **Table 2.** Clinical Significance Test - Region A. **Table 3.** Clinical Significance Test - Region B.

**Additional file 4.** Facility Assessment Results.

**Additional file 5.** Qualitative Analysis: Themes by Participant.

## Data Availability

The datasets generated during and/or analysed during the current study are available from the corresponding author on reasonable request.

## Abbreviations

HCW: Health Care Workers
LMICs: Low- and Middle-Income Countries
SP: Sterile Processors
SPECT: Sterile Processing Education Charitable Trust
SSI: Surgical Site Infection
