# Supplementary material for: The impact of a sterile processing program in Northwest Tanzania: a mixed-methods study
Source: Antimicrob Resist Infect Control. 2019 Nov 20;8:183. doi: 10.1186/s13756-019-0633-0 (PMC6868803; doi:10.1186/s13756-019-0633-0)
Supplement: Supplementary file 2 — Additional file 2. SPECT – Participant Test. [file 13756_2019_633_MOESM2_ESM.doc]

SPECT – Participant Test

Name: ___________________________ Date: ___________________________

1. Which of the following statements about microorganisms is true?
   1. All microorganisms are harmful to humans
   2. Healthy humans do not carry microorganisms
   3. Microorganisms are necessary for life
   4. Microorganisms die within 10 minutes of leaving the body
2. When soil remains on a device after cleaning:
   1. The device will be difficult to sterilize
   2. The device cannot be sterilized
   3. The device will be safe to use
   4. It will take longer to cool after sterilization
3. How frequently should the sterilizer chamber be thoroughly cleaned?
   1. Daily
   2. After each use
   3. Once weekly
   4. When the sterilizer’s operating control gauge indicates that cleaning is necessary
4. The main reason standard precautions are practiced is that:
   1. All patients are infectious
   2. Patients diagnosed with a specific disease may be infectious
   3. Patients are generally healthy unless they show symptoms of an infectious disease
   4. Patients may pose a risk of infection whether they have been diagnosed with an infectious disease or not
5. Watches and other jewelry should not be worn in the sterilization work areas because:
   1. They harbor bacteria
   2. They are expensive
   3. They may be damaged
   4. They may be misplaced or stolen
6. Which of the following is not an example of surgical asepsis?
   1. Sterilization of instruments
   2. Operating room dress codes
   3. Cleaning the instruments with water and detergent
   4. Operating room techniques to prevent contamination of sterile instruments
7. Floors in the sterilization area should be:
   1. Wet-mopped daily
   2. Wet-mopped weekly
   3. Swept daily and wet-mopped weekly
   4. Swept daily
8. Microorganisms reproduce every 20 minutes by a process called:
   1. Repopulation
   2. Binary fission
   3. Replication
   4. Bilateral reproduction
9. The absence of microorganisms that produce disease is called:
   1. Infection prevention
   2. Infection control
   3. Asepsis
   4. HIA-control
10. The most efficient type of dry heat sterilizer is:
    1. The gravity convection sterilizer
    2. The mechanical convection sterilizer
    3. The dynamic air removal sterilizer
    4. The special purpose dry heat oven
11. Bacteria that grow well at body temperature and often cause illness in humans are:
    1. Psychrophiles
    2. Mesophiles
    3. Thermophiles
    4. Aerobicides
12. Sterile packages should be stored higher than _________ centimeters from the floor.
    1. 5 – 10 cm
    2. 10 – 15 cm
    3. 20 – 25 cm
    4. 30 – 35 cm
13. To achieve a reasonable level of disinfection, alcohol used as an intermediate-level disinfectant must remain in wet contact with the surface of the object being disinfected for a minimum of __________ minutes.
    1. 2
    2. 3
    3. 4
    4. 5
14. A mode of bacterial transmission that would take place when an infectious agent is transmitted through an item such as food or water is called:
    1. Contact
    2. Common vehicle
    3. Airborne
    4. Vector-borne
15. Bacteria that grow well in warm temperatures (50°C - 70°C) are called:
    1. Aerobic
    2. Anaerobic
    3. Thermophiles
    4. Mesophiles
16. These chemicals are used to slow the growth of bacteria on skin and cannot be used to disinfect medical devices.
    1. Disinfectants
    2. Surfactants
    3. Chelating agents
    4. Antiseptics
17. The two most common temperatures used in steam sterilization are:
    1. 50°C and 60°C - 63°C
    2. 38°C and 55°C – 58°C
    3. 160°C and 163°C
    4. 121°C and 132°C – 134°C
18. The first step in the sterilization process is:
    1. Placing instruments in the sterilizer
    2. Disinfecting the instruments
    3. Thorough cleaning of instruments
    4. Proper packaging of instruments
19. Some bacteria develop hard shells around them, which make them more difficult to destroy. Those bacteria are called:
    1. Spores
    2. Bacilli
    3. Pyrogenic
    4. Cells
20. To elevate the temperature in a steam sterilizer, you must:
    1. Increase the steam pressure
    2. Decrease the steam pressure
    3. Allow air inside
    4. All of the above
21. An infectious agent which grows only in living tissue is called:
    1. A germ
    2. A virus
    3. Pseudomonas
    4. Protoplasm
22. What would be the recommended exposure time for a Dry Heat Sterilization load that was run at an exposure temperature of 160°C?
    1. 1 hour
    2. 1.5 hours
    3. 2 hours
    4. 2.5 hours
23. How frequently should a steam sterilizer’s door gasket be inspected and cleaned?
    1. Daily
    2. After each use
    3. Once weekly
    4. When the machine’s operating control gauge indicates there is a major air leak.
24. When washing your hands, sterilization technicians should actively scrub with soap and water for minimum of ____________ seconds.
    1. 20
    2. 30
    3. 40
    4. 50
25. A chemical which kills most pathogenic organisms but does not kill spores is called:
    1. Bacterial
    2. An antiseptic
    3. A disinfectant
    4. None of the above
26. In which area of the hospital should soiled instruments and other contaminated items be received?
    1. Preparation Area
    2. Packaging Area
    3. Decontamination Area
    4. Sterilization Area
27. Which of the following is not the definition of decontamination?
    1. To make safe by removing or reducing contamination by infectious organisms
    2. The reduction of contamination to an acceptable level
    3. A process by which all forms of microbial life including bacteria, viruses, spores and fungi are destroyed
    4. None of the above
28. The term “critical devices” refers to objects that:
    1. Are introduced directly into the bloodstream or other sterile areas of the body
    2. Come in contact with unbroken skin, such as blood pressure cuffs
    3. Come in contact with mucous membranes
    4. All of the above
29. Filtered water should be used:
    1. As the first rinse water in the cleaning process
    2. To soften water for removal of calcium and magnesium
    3. As the last rinse water during the manual cleaning of instruments
    4. At all times when cleaning
30. ______________ is the first step in reprocessing a medical device after it’s been used:
    1. Disinfecting
    2. Cleaning
    3. Sanitizing
    4. Sterilizing
31. The most common way for microorganisms to spread through the hospital is:
32. Hands
33. Poor ventilation
34. Brushes used for cleaning
35. Unsterile surgical instruments
36. How do you know if a surgical instrument is safe to use for a surgical procedure?
37. There is no visible soil
38. It has been inspected and is working properly
39. It has been sterilized at the appropriate time and temperature
40. All of the above
41. Which sentence is TRUE regarding disinfectants?
    1. Disinfectant solutions do not cause surgical instruments to rust
    2. Diluting disinfectants can increase resistant microorganisms
    3. The longer the tools soak in disinfectant the more bacteria are killed
    4. Disinfectants eliminate all microorganisms
42. What is an important step to remember when transporting contaminated items?
    1. Transport containers should be labeled to indicate biohazardous contents
    2. Contaminated instruments must be covered to prevent the spread of microorganisms
    3. Contaminated surgical tools should be transported as soon as possible to prevent blood and other soil from drying on them
    4. All of the above
43. Choose the preferred sterilization method for surgical instruments:
    1. Dry Heat Sterilization
    2. High-Level Disinfection
    3. Steam Sterilization
    4. All of the above
44. Sterilized items are considered unsterile if they are:
    1. Wet
    2. Dusty
    3. Dropped on the ground
    4. All of the above
45. You can break the Chain of Infection by:
    1. Washing your hands
    2. Disinfecting a medical device
    3. Sterilizing surgical instruments
    4. All of the above
46. What temperature does the Dry Heat Sterilizer need to be set at to kill resistant microorganisms?
    1. 70°C
    2. 80°C
    3. 100°C
    4. Above 120°C
47. What category do surgical instruments and implants fall under?
    1. Semi-critical
    2. Critical
    3. Non-critical
    4. All of the above
48. When preparing and packaging instruments for steam sterilization, the instruments must be:
    1. Completely assembled and in locked position
    2. Arranged in alphabetical order
    3. Completely disassembled and in open position
    4. Arranged in order from the smallest instrument to the largest
