# Supplementary material for: The impact of a sterile processing program in Northwest Tanzania: a mixed-methods study
Source: Antimicrob Resist Infect Control. 2019 Nov 20;8:183. doi: 10.1186/s13756-019-0633-0 (PMC6868803; doi:10.1186/s13756-019-0633-0)
Supplement: Supplementary file 3 — Additional file 3. Statistical Analysis. Table 1. Hypotheses Testing. Table 2. Clinical Significance Test - Region A. Table 3. Clinical Significance Test - Region B. [file 13756_2019_633_MOESM3_ESM.docx]

Appendix III –Statistical Analysis

**Table 1 - Hypotheses Testing**

**Table 2 - Clinical Significance Test - Region A**

**Table 3 - Clinical Significance Test - Region B**

| **Region B** | | | Cohen's test (pre-post1) | | | Cohen's test (pre-post2) | | |  |
| --- | --- | --- | --- | --- | --- | --- | --- | --- | --- |
| Pre-Train Test | Post-Train Test 1 | Post Train  Test 2 | d | MCID | Clinical effect | d | MCID | Clinical effect | %Δ_MCID_ |
| 8 | 19 | 16 | 11 | 2.79 | large(+) | 8 | 2.03 | large(+) | -27.27 |
| 13 | 23 | 13 | 10 | 2.54 | large(+) | 0 | 0.00 | no effect | -100.00 |
| 10 | 17 | 15 | 7 | 1.78 | large(+) | 5 | 1.27 | large(+) | -28.57 |
| 13 | 19 | 15 | 6 | 1.52 | large(+) | 2 | 0.51 | moderate(+) | -66.67 |
| 12 | 19 | 14 | 7 | 1.78 | large(+) | 2 | 0.51 | moderate(+) | -71.43 |
| 12 | 18 | 20 | 6 | 1.52 | large(+) | 8 | 2.03 | large(+) | 33.33 |
| 16 | 21 | 19 | 5 | 1.27 | large(+) | 3 | 0.76 | moderate(+) | -40.00 |
| 9 | 12 | 11 | 3 | 0.76 | moderate(+) | 2 | 0.51 | moderate(+) | -33.33 |
| 10 | 21 | 21 | 11 | 2.79 | large(+) | 11 | 2.79 | large(+) | 0.00 |
| 13 | 18 | 7 | 5 | 1.27 | large(+) | -6 | -1.52 | large(-) | -220.00 |
| 9 | 15 | 15 | 6 | 1.52 | large(+) | 6 | 1.52 | large(+) | 0.00 |
| 13 | 18 | 16 | 5 | 1.27 | large(+) | 3 | 0.76 | moderate(+) | -40.00 |
| 8 | 14 | 11 | 6 | 1.52 | large(+) | 3 | 0.76 | moderate(+) | -50.00 |
| 5 | 15 | 8 | 10 | 2.54 | large(+) | 3 | 0.76 | moderate(+) | -70.00 |
| 14 | 21 |  | 7 | 1.78 | large(+) | - | - | - | - |
| 8 | 14 | 12 | 6 | 1.52 | large(+) | 4 | 1.01 | large(+) | -33.33 |
| 6 | 15 | 18 | 9 | 2.28 | large(+) | 12 | 3.04 | large(+) | 33.33 |
| 11 | 15 | 16 | 4 | 1.01 | large(+) | 5 | 1.27 | large(+) | 25.00 |
