# Supplementary material for: The impact of a sterile processing program in Northwest Tanzania: a mixed-methods study
Source: Antimicrob Resist Infect Control. 2019 Nov 20;8:183. doi: 10.1186/s13756-019-0633-0 (PMC6868803; doi:10.1186/s13756-019-0633-0)
Supplement: Supplementary file 4 — Additional file 4. Facility Assessment Results. [file 13756_2019_633_MOESM4_ESM.docx]

**Appendix IV**

**Facility Assessment Results**

|  | **Region A** | | | **Region B** | | | **Total Pre/Post** |
| --- | --- | --- | --- | --- | --- | --- | --- |
| **Results of 13 SP areas assessed in both regions** | (Yes)  Pre (post) | (No)  Pre  (post) | (n/a) | (Yes) | (No) | (n/a) |  |
| General Info | | | |  |  |  |  |
| SP area cleaned 1X/day | 8(8) | 0(0) | 0 | 5(5) | 0 | 0 | 13/13 |
| SP area cleaned thoroughly 1X/wk | 7(8) | 1(0) | 0 | 5(5) | 0 | 0 | 12/12 |
| There are signs restricting access of unauthorized personnel in the SP areas | 5(5) | 3(3) | 0 | 3(5) | 2(0) | 0 | 8/10 |
| Point of Use Preparation | | | |  |  |  |  |
| Instruments are wiped during the surgical procedure to remove visible blood | 0(6) | 8(2) | 0 | 1(3) | 4(2) | 0 | 1/9 |
| Instruments are soaked in water and detergent after use to loosen soil | 1(6) | 7(2) | 0 | 0(2) | 5(3) | 0 | 1/8 |
| Sharp instruments are separated from other instruments to minimize risk of injury to the cleaner | 7(7) | 1(1) | 0 | 5(5) | 0 | 0 | 12/12 |
| Multi-part instruments are disassembled before sending to dirty area | 3(5) | 2(0) | 3(3) | 2(2) | 2(2) | 1(1) | 5/7 |
| Hinged instruments are placed in open position with ratchets unlocked | 6(8) | 2(0) | 0 | 1(3) | 3(1) | 1(1) | 7/11 |
| Delicate instruments are protected | 0(1) | 1(0) | 7(7) | 0(1) | 1(0) | 4(4) | 0/2 |
| Disposable sharps are placed in a sharps bin at point of use | 8(8) | 0 | 0 | 5(5) | 0 | 0 | 13/13 |
| Transport of items from Dirty to Clean | | | |  |  |  |  |
| Contaminated instruments flow from dirty to clean areas during transportation without cross contamination | 1(1) | 7(7) | 0 | 2(4) | 3(1) | 0 | 3/5 |
| Contaminated items are placed in an enclosed container during transport from the OR to the dirty area | 1(6) | 7(2) | 0 | 0(3) | 5(2) | 0 | 1/9 |
| Contaminated instruments are transported to the dirty area immediately after use | 8(8) | 0 | 0 | 5(5) | 0 | 0 | 13/13 |
| Manual Cleaning in Dirty Area | | | |  |  |  |  |
| The dirty area is physically separate from the inspection/packaging area | 6(7) | 2(1) | 0 | 5(4) | 0(1) | 0 | 11/11 |
| The following types of PPE are worn by HCW while cleaning instruments: |  | | |  |  |  |  |
| gloves | 8(8) | 0 | 0 | 5(5) | 0 | 0 | 13/13 |
| gowns | 8(8) | 0 | 0 | 5(5) | 0 | 0 | 13/13 |
| head covers | 7(6) | 1(2) | 0 | 4(5) | 1(0) | 0 | 11/11 |
| protective eye wear | 7(7) | 1(1) | 0 | 1(3) | 4(2) | 0 | 8/11 |
| masks | 7(8) | 1(0) | 0 | 5(5) | 0 | 0 | 12/13 |
| dedicated shoes | 8(8) | 0 | 0 | 5(5) | 0 | 0 | 13/13 |
| Instruments are 'decontaminated' in 0.5% chlorine solution* | 8(3) | 0(5) | 0 | 4(1) | 1(4) | 0 | 12/4* |
| Visible gross soil, such as blood, is removed from the instruments within 1 hr after use | 8(8) | 0 | 0 | 5(5) | 0 | 0 | 13/13 |
| Medium sized nail brushes are used to clean instruments | 0(0) | 8(8) | 0 | 0(4) | 5(1) | 0 | 0/4 |
| Small toothbrush style brushes are available to clean instruments | 1(6) | 7(2) | 0 | 0(5) | 5(0) | 0 | 1/11 |
| Enzymatic cleaning detergents are available and used to clean instruments | 0(0) | 8(8) | 0 | 0 | 5(5) | 0 | 0/0 |
| Instruments are cleaned using 3 buckets/sinks (1st for soap & water, 2nd for soap & water, 3rd for clean water) | 0(7) | 8(1) | 0 | 0(5) | 5(0) | 0 | 0/12 |
| Instruments are carefully inspected at each step of the cleaning process | 4(5) | 4(3) | 0 | 3(3) | 2(2) | 0 | 6/7 |
| Instruments are scrubbed below the surface of the water | 3(6) | 5(2) | 0 | 1(5) | 4(0) | 0 | 4/11 |
| Instruments are in unlocked position while they're being cleaned | 8(8) | 0 | 0 | 5(4) | 0(1) | 0 | 13/12 |
| Chlorine is used as a high-level disinfectant on medical devices | 4(4) | 3(3) | 1(1) | 2(1) | 3(4) | 0 | 6/5* |
| Alcohol is used as a high-level disinfectant on medical devices | 3(0) | 4(7) | 1(1) | 2(0) | 3(5) | 0 | 5/0* |
| Glutaraldyhyde is used as a high-level disinfectant on medical devices | 2(1) | 5(6) | 1(1) | 2(3) | 3(2) | 0 | 4/4 |
| Formaldehyde is used as a high-level disinfectant on medical devices | 0 | 7(7) | 1(1) | 0 | 5(5) | 0 | 0/0 |
| Inspection, Assembly & Packaging in Clean Area | | | |  |  |  |  |
| After instruments have been cleaned, they are moved to a clean table or separate room away from the dirty area | 5(7) | 3(1) | 0 | 5(5) | 0 | 0 | 10/12 |
| Instruments are thoroughly dried with a clean cloth | 4(5) | 4(3) | 0 | 3(0) | 2(5) | 0 | 7/5 |
| Each individual instrument is carefully inspected prior to packaging | 5(8) | 3(0) | 0 | 5(5) | 0 | 0 | 10/13 |
| Instruments are function tested prior to packaging | 7(8) | 1(0) | 0 | 5(5) | 0 | 0 | 12/13 |
| Instruments are packaged with a protective cover, such as cloth, or placed inside a rigid container | 5(6) | 3(2) | 0 | 5(5) | 0 | 0 | 10/11 |
| After the instruments are packaged, the package is labeled | 6(5) | 2(3) | 0 | 5(5) | 0 | 0 | 11/10 |
| Sterilization | | | |  |  |  |  |
| A functioning steam sterilizer is used to sterilize instruments | 4(6) | 4(2) | 0 | 5(5) | 0 | 0 | 9/11 |
| A functioning dry heat sterilizer is used to sterilize instruments | 4(5) | 4(3) | 0 | 0 | 5(5) | 0 | 4/5 |
| Sterilization tape is being used on the outside instrument packages | 4(6) | 4(2) | 0 | 1(4) | 4(1) | 0 | 5/10 |
| Chemical Indicators (Cis) are being placed inside instrument packages | 0(4) | 8(4) | 0 | 0(2) | 5(3) | 0 | 0/6 |
| A record sheet is used to track the instruments going in and coming out of the sterilizer | 1(1) | 7(7) | 0 | 0(2) | 5(3) | 0 | 1/3 |
| Sterile Storage | | | |  |  |  |  |
| After sterilization, instruments are stored in an enclosed cabinet or designated storage room | 3(6) | 5(2) | 0 | 5(5) | 0 | 0 | 8/11 |
| Sterilized packages are labeled with an expiry date | 5(6) | 3(2) | 0 | 5(5) | 0 | 0 | 10/11 |
