# Supplementary material for: The impact of a sterile processing program in Northwest Tanzania: a mixed-methods study
Source: Antimicrob Resist Infect Control. 2019 Nov 20;8:183. doi: 10.1186/s13756-019-0633-0 (PMC6868803; doi:10.1186/s13756-019-0633-0)
Supplement: Supplementary file 4 [file 13756_2019_633_MOESM1_ESM.docx]

**SPECT Sterile Processing (SP) Facility Assessment Tool**

*** This assessment form should be filled out for each hospital department providing cleaning an**

**Date (yy/mm/dd): ___________________________________________**

**Name of Hospital: _____________________________________________________________**
**Hospital Director: _____________________________________________________**
**Contact info: ______________________________________________________________________**

**Head of Department: ____________________________________________________________**
**Job Title: __________________________________________________________________________**
**Contact Info: ______________________________________________________________________**
**Name(s) and info of any other staff member working in SP:**

| **Hospital and Staff Information** | **Nu** |
|---|---|
| Average number of surgical procedures performed weekly in this department | |
| Number of staff who clean instruments in this department | |
| Number of staff who inspect and package instruments | |
| Number of staff who sterilize instruments | |
| Number of staff who have received training in cleaning and sterilization | |
| Types of procedures performed in this department: | |
| Staff that primarily clean the instruments (ie nurses or designated): | |

| **General Info** | **Ye** |
|---|---|
| The SP areas are cleaned once a day by house keeping staff | |
| The SP areas are *thoroughly* cleaned once a week by SP staff | |
| There are signs restricting access of unauthorized personel in the SP areas | |

| **Point of Use Preparation** | **Ye** |
|---|---|

| | Yes | No | |
|---|---|---|---|
| Multi-part instruments are disassembled before sending to dirty area | | | |
| Hinged instruments are placed in open position with ratchets unlocked | | | |
| Delicate instruments are protected | | | |
| Disposable sharps are placed in a sharps bin at point of use | | | **Comment:** |

| **Transport of items from Dirty to Clean** | **Yes** | **No** | |
|---|---|---|---|
| Contaminated instruments flow from dirty to clean areas during transportation ***without*** cross contamination | | | |
| Contaminated items are placed in an enclosed container during transport from the OR to the dirty area | | | |
| Contaminated instruments are transported to the dirty area immediately after use | | | **Comment:** |

| **Manual Cleaning in Dirty Area** | **Yes** | **No** | |
|---|---|---|---|
| The dirty area is physically separate from the inspection/packaging area | | | |
| The following types of PPE are worn by staff while cleaning instruments: | | | |
| gloves | | | |
| gowns | | | |
| head covers | | | |
| protective eye wear | | | |
| masks | | | |
| dedicated shoes | | | |
| Instruments are 'decontaminated' in 0.5% chlorine solution | | | |
| Visible gross soil, such as blood, is removed from the instruments within 1 hr after use | | | |
| Medium sized nail brushes are used to clean instruments | | | |
| Small toothbrush style brushes are available to clean instruments | | | |
| Enzymatic cleaning detergents are available and used to clean instruments | | | |
| Instruments are cleaned using 3 buckets/sinks (1st for soap & water, 2nd for soap & water, 3rd for clean water) | | | |
| Instruments are carefully inspected at each step of the cleaning process | | | |
| Instruments are scrubbed below the surface of the water | | | |
| Instruments are in unlocked position while they're being cleaned | | | |
| Chlorine is used as a high-level disinfectant on medical devices | | | |
| Alcohol is used as a high-level disinfectant on medical devices | | | |
| Glutaraldyhyde is used as a high-level disinfectant on medical devices | | | |
| Formaldehyde is used as a high-level disinfectant on medical devices | | | **Comment:** |

| **Inspection, Assembly & Packaging in Clean Area** | **Yes** | **No** | |
|---|---|---|---|
| After instruments have been cleaned, they are moved to a clean table or separate room away from the dirty area | | | |
| Instruments are thoroughly dried with a clean cloth | | | |
| Each individual instrument is carefully inspected prior to packaging | | | |
| Instruments are function tested prior to packaging | | | |
| Instruments are packaged with a protective cover, such as cloth, or placed inside a rigid container | | | |
| After the instruments are packaged, the package is labeled | | | **Comment:** |

| **Sterilization** | **Yes** | **No** | |
|---|---|---|---|
| A functioning steam sterilizer is used to sterilize instruments | | | |
| A functioning dry heat sterilizer is used to sterilize instruments | | | |
| Sterilization tape is being used on the outside instrument packages | | | |
| Chemical Indicators (Cis) are being placed inside instrument packages | | | |
| A record sheet is used to track the instruments going in and coming out of the sterilizer | | | |
| Length of time and temperatures used to sterilize instruments is: | | | **Comment:** |

| **Sterile Storage** | **Yes** | **No** | |
|---|---|---|---|
| After sterilization, instruments are stored in an enclosed cabinet or designated storage room | | | |
| Sterilized packages are labeled with an expiry date | | | **Comment:** |
