# Supplementary material for: The impact of a sterile processing program in Northwest Tanzania: a mixed-methods study
Source: Antimicrob Resist Infect Control. 2019 Nov 20;8:183. doi: 10.1186/s13756-019-0633-0 (PMC6868803; doi:10.1186/s13756-019-0633-0)
Supplement: Supplementary file 5 [file 13756_2019_633_MOESM5_ESM.docx]

Appendix V– Qualitative Analysis: Themes by Participant

Participant	Changes in practice	Perceived changes in SSI incidence rates	Reciprocal relationship and partnership building	Challenges in implementing practice changes	Resource constraints	Influence of formalized policy and guidelines	Personal and professi[illegible] growth
**Region A**							
1	✓			✓	✓		✓
2	✓			✓	✓	✓	✓
9	✓				✓		✓
10	✓	✓	✓	✓	✓	✓	✓
11	✓			✓	✓		✓
12	✓	✓		✓	✓	✓	✓
20	✓			✓		✓	✓
21	✓			✓		✓	✓
22	✓			✓	✓	✓	✓
23	✓			✓	✓	✓	✓
25	✓			✓	✓	✓	✓
26	✓	✓	✓	✓	✓	✓	✓
27	✓			✓	✓	✓	✓
28	✓	✓		✓	✓	✓	✓
29	✓			✓	✓		✓
30	✓			✓	✓		
31	✓			✓	✓		✓
38	✓			✓	✓	✓	✓
**Region B**							
1	✓			✓	✓		✓
2	✓						✓
3	✓	✓		✓	✓		✓
4	✓		✓	✓	✓		✓
5	✓		✓	✓	✓		✓
6	✓		✓	✓	✓	✓	
7	✓			✓	✓		
8	✓		✓		✓		✓
10	✓		✓	✓	✓		
11	✓		✓	✓	✓		
12	✓		✓	✓			
13	✓	✓		✓	✓		✓
14	✓		✓	✓	✓		✓
